# Construction and comprehensive analysis of a curoptosis-related lncRNA signature for predicting prognosis and immune response in cervical cancer

**DOI:** 10.3389/fgene.2023.1023613

**Published:** 2023-01-27

**Authors:** Li Liu, Jianfeng Zheng, Hongmei Xia, Qiaoling Wu, Xintong Cai, Liyan Ji, Yang Sun

**Affiliations:** ^1^ Department of Gynecology, Clinical Oncology School of Fujian Medical University, Fujian Cancer Hospital, Fuzhou, China; ^2^ Department of Gynecology, Fujian Cancer Hospital, Fuzhou, China; ^3^ Geneplus-Beijing Institute, Beijing, China

**Keywords:** cervical cancer, cuproptosis, lncRNA, prognosis, radiotherapy, immune

## Abstract

Cuproptosis (copper-ion-dependent cell death) is an unprogrammed cell death, and intracellular copper accumulation, causing copper homeostasis imbalance and then leading to increased intracellular toxicity, which can affect the rate of cancer cell growth and proliferation. This study aimed to create a newly cuproptosis-related lncRNA signature that can be used to predict survival and immunotherapy in patients with cervical cancer, but also to predict prognosis in patients treated with radiotherapy and may play a role in predicting radiosensitivity. First of all, we found lncRNAs associated with cuproptosis between cervical cancer tumor tissues and normal tissues. By LASSO-Cox analysis, overlapping lncRNAs were then used to construct lncRNA signatures associated with cuproptosis, which can be used to predict the prognosis of patients, especially the prognosis of radiotherapy patients, ROC curves and PCA analysis based on cuprotosis-related lncRNA signature and clinical signatures were developed and demonstrated to have good predictive potential. In addition, differences in immune cell subset infiltration and differences in immune checkpoint expression between high-risk and low-risk score groups were analyzed, and we investigated the relationship between this signature and tumor mutation burden. In summary, we constructed a lncRNA prediction signature associated with cuproptosis. This has important clinical implications, including improving the predictive value of cervical cancer patients and providing a biomarker for cervical cancer.

## Background

Cervical cancer (CC) poses a genuine threat to women’s health. CC is the most common malignant tumor in women worldwide. In recent years, owing to advances in surgery, radiotherapy/radiochemotherapy, and the widespread use of early CC screening, the incidence of CC has decreased significantly in developed countries. In 2020, the World Health Organization implemented a global strategy aimed at reducing the incidence of CC to four cases per 100,000 women by the 21st century (Wilailak et al., 2021). The majority of new cases (∼85%) and deaths (∼90%) worldwide occur in low- and middle-income countries (Bhatla et al., 2021). Although secondary prevention through new vaccinations and primary prevention through cancer screening is common, in 2014, only 1% of girls from low- and middle-income countries were vaccinated globally (Sundstrom and Elfstrom, 2020). Human papillomavirus (HPV) infection contributes to the development of CC (Munoz et al., 2003), however, its impact on CC prognosis is currently inconclusive, and radiotherapy is the main treatment for patients with advanced CC. Moreover, the response to radiotherapy is indicative of the prognosis of patients with CC. Therefore, an ideal predictive model or accurate prognostic biomarker to guide CC treatment is warranted.

Heavy metal ions are essential micronutrients, however, their excessive or insufficient consumption can kill cells. Different subprogrammes can cause regulated cell death in response to stress caused by heavy metals. For example, ferroproteinases can cause iron-dependent types of oxidative cell death due to unrestricted lipid peroxidation (Tang et al., 2022). ‘Cuproptosis’, a non-apoptotic, novel type of cell death, was recently discovered by Tzvitkov et al. (Li et al., 2022). They noted that copper causes cell death primarily by binding to the lipoacylated components of the tricarboxylic acid cycle, which in turn leads to toxic protein stress and cell death (Tsvetkov et al., 2022).

Long non-coding RNAs (lncRNAs) are non-protein-coding RNA molecules whose transcripts are >200 nucleotides in length (Gibb et al., 2011; Zhang et al., 2020), these were originally thought to be by-products of RNA polymerase II transcription (Mercer et al., 2009). By interfering with DNA, miRNAs, and proteins, lncRNAs play an important role in the occurrence and development of tumors (Wang and Chang, 2011; Yoon et al., 2012; Li et al., 2019), inducing differences in the expression of key genes (Ma et al., 2019; Shahabi et al., 2019). Increasing research has suggested that lncRNAs function in many types of malignant tumors, affecting cell differentiation, growth, invasion, migration, and apoptosis as well as the cell cycle and tumor’s resistance to chemotherapy (Qian et al., 2021; Zhang et al., 2021; Chen et al., 2022). To date, cuproptosis-related lncRNAs have been studied in renal tumors (Xu et al., 2022a), liver tumors (Zhang et al., 2022), soft tissue sarcomas (Han et al., 2022), *etc.* However, the mechanisms through which copper regulates tumor cell death remain unclear, and there is no conclusive evidence that cuproptosis-related lncRNAs are associated with CC. We hope that the characteristics of the cuproptosis-related lncRNAs engineered herein will provide help predict CC prognosis and the mechanism of cuproptosis in CC.

## Materials and methods

### Public data collection and processing

The Cancer Genome Atlas (TCGA) database (https://portal.gdc.cancer.gov/) was used to retrieve the RNA sequencing, mutation, and clinical data of 307 samples (304 CC samples and three normal samples) from 73 patients who received radiotherapy and provided post-radiotherapy evaluation information. The Perl programming language (https:// www.perl.org, version Strawberry-Perl-5.30.0) was used to process the data. The transcriptome and clinical data were pre-processed to extract the expression profile matrix of the coding genes and lncRNAs and the clinical and pathological characteristics of the patients with CC, including their survival status, survival times, CC stage and grade, and TMN. We performed a literature search (Ge et al., 2022; Li et al., 2022; Tang et al., 2022; Tsvetkov et al., 2022) and found fourteen genes associated with cuproptosis (*FDX1*, *ATP7B*, *GCSH*, *DBT*, *GLS*, *CDKN2A*, *MTF1*, *SLC31A1*, *PDHB*, *LIAS*, *DLD*, *LIPT1*, *DLAT*, and *PDHA1*).

### Screening of cuproptosis-related lncRNAs

We used the Pearson correlation analysis to assess the correlation between cuproptosis-related lncRNAs and mRNAs (|corFilter = 0.4|; pvalueFilter = 0.001) in R software using the ‘limma,’ ‘ggplot2’, ‘ggalluvial’, and ‘dplyr’ packages. Finally, Sankey plots were drawn to present the correlations between cuproptosis-related lncRNAs and all of the 14 genes.

### Construction of prognostic cuproptosis-related lncRNA signatures

The R package ‘caret’ was used to randomly divide the cervical squamous cell carcinoma and endocervical adenocarcinoma (CESC) datasets retrieved from TCGA into the training and testing sets. The signature was validated using the testing set and the entire TCGA dataset.

The lncRNAs related with cuproptosis were screened using univariate Cox regression analysis (*p* < 0.05) and the results were presented using forest plots. In addition, the Lasso-Cox regression analysis was used to determine the optimal prognostic lncRNA group, and a risk model was established (penalty parameters were estimated through 1000-fold cross-validation); this approach minimized overfitting in the modeling process. Finally, multivariate Cox regression analysis was used to establish a prognostic model based using the best lncRNAs; three lncRNAs were selected for model construction, after which a heat map of the correlation between the three lncRNAs and cuproptosis-related genes was plotted using the ‘ggplot2’ package. The following equations were applied to each patient with CC to assess the risk score:

Risk score=ΣβlncRNA×Exp lncRNA

Based on the risk score median value, the patients were divided into the high-risk group (HRG) and the low-risk group (LRG). Kaplan–Meier curves were generated to determine whether the overall survival (OS) and progression-free survival (PFS) differed between the HRG and LRG. We also verified the independence of the model with an independent analysis approach using factors such as patient age and CC grade, and stage. Based on the patient’s survival, and the survminer and timeROC programs, receiver operating characteristic (ROC) curves were generated, and the area under the curve (AUC) was calculated. To measure the model’s accuracy, the concordance index (C-index) was used. A heat map was created to illustrate the correlation between the model and clinical characteristics. In the 73 patients with CC who underwent radiotherapy, the survival rate differences between different risk groups were analyzed, and the lncRNAs involved in constructing the model were further investigated to understand the differences in their expressions between the radiosensitive and insensitive groups.

### Nomogram and principal component analysis

Using the ‘rms’ and ‘survival’ packages, we created line graphs for patients with CC having 1-, 3-, and 5-year OS, with the risk scores combined with their clinicopathologic factors. Using Hosmer–Lemeshow calibration curves, we tested whether the developed nomogram had any predictive power. Principal component analysis (PCA) was conducted using the ‘scatterplot3d’ package in the R program. PCA was also used to categorize the expression patterns of the four cohorts–all the genes, cuproptosis-related genes, cuproptosis-related LncRNAs, and the three lncRNA signatures (risk DE-lncRNAs)–to display the spatial distribution of the high-risk and low-risk samples.

### Gene ontology and kyoto encyclopaedia of genes and genomics analysis

Through differential gene screening, we identified 173 genes associated with HRGs and LRGs in 304 patients. We then performed Gene Ontology (GO) analysis for assessing biological processes, cellular processes, and molecular functions. We also used GO analysis to analyze differentially expressed Kyoto Encyclopaedia of Genes and Genomics (KEGG) pathways in both groups with the ‘clusterProfiler’, ‘org.Hs.eg.db’, ‘enrichplot’, and ‘circlize’ packages. The enriched biosynthetic pathways and processes with a *p*-value of <0.05 and a FDR of <0.05 were considered statistically significant.

### Immune infiltration analysis

We investigated immune infiltration and immune-related functions in CC using single-sample gene set enrichment analysis with ‘limma’, ‘GSVA’, and ‘GSEABase’ packages. To investigate the relationship between this feature and the immune infiltration status, we used six algorithms (TIMER, CIBERSORT, CIBERSORT-ABS, QUANTISEQ, MCPCOUNTER, and XCELL) to calculate the immune infiltration profile of the TCGA–CESC dataset and present the results as a heat map. Based on the estimation algorithm, we then calculated the percentage of tumor-infiltrating immune cells in the CC microenvironment associated with the three lncRNA signatures. Furthermore, we investigated the differences in the expressions of immune checkpoints in the HRG and LRG and presented the results as boxplots.

### Differences in tumor mutation burden survival

After downloading the somatic mutation data, mutation information was extracted from the TCGA database using the Perl programming language. The ‘maftools’ package in R software was used to analyze differences in the mutations between the HRG and LRG. Using the ‘survminer’ package for survival analysis, we then examined and integrated the mutations with clinical data and analyzed differences in survival across mutation scenarios after combining the results of HRG and LRG.

### Statistical analysis

The Perl programming language was used to extract and integrate the data, whereas the R program (version 4.2.1) was used for the Lasso-Cox regression, survival, and PCA analyses. Kaplan-Meier analysis was performed using the ‘Survival’ package. In addition, the ‘survival’, ‘survivor’, ‘survival’, ‘pheatmap’, and ‘ggpubr’ software packages were used to validate the prediction model. *p* < 0.05 was considered statistically significant.

## Results

### Construction of the cuproptosis-related lncRNA signature

**FIGURE 1:**
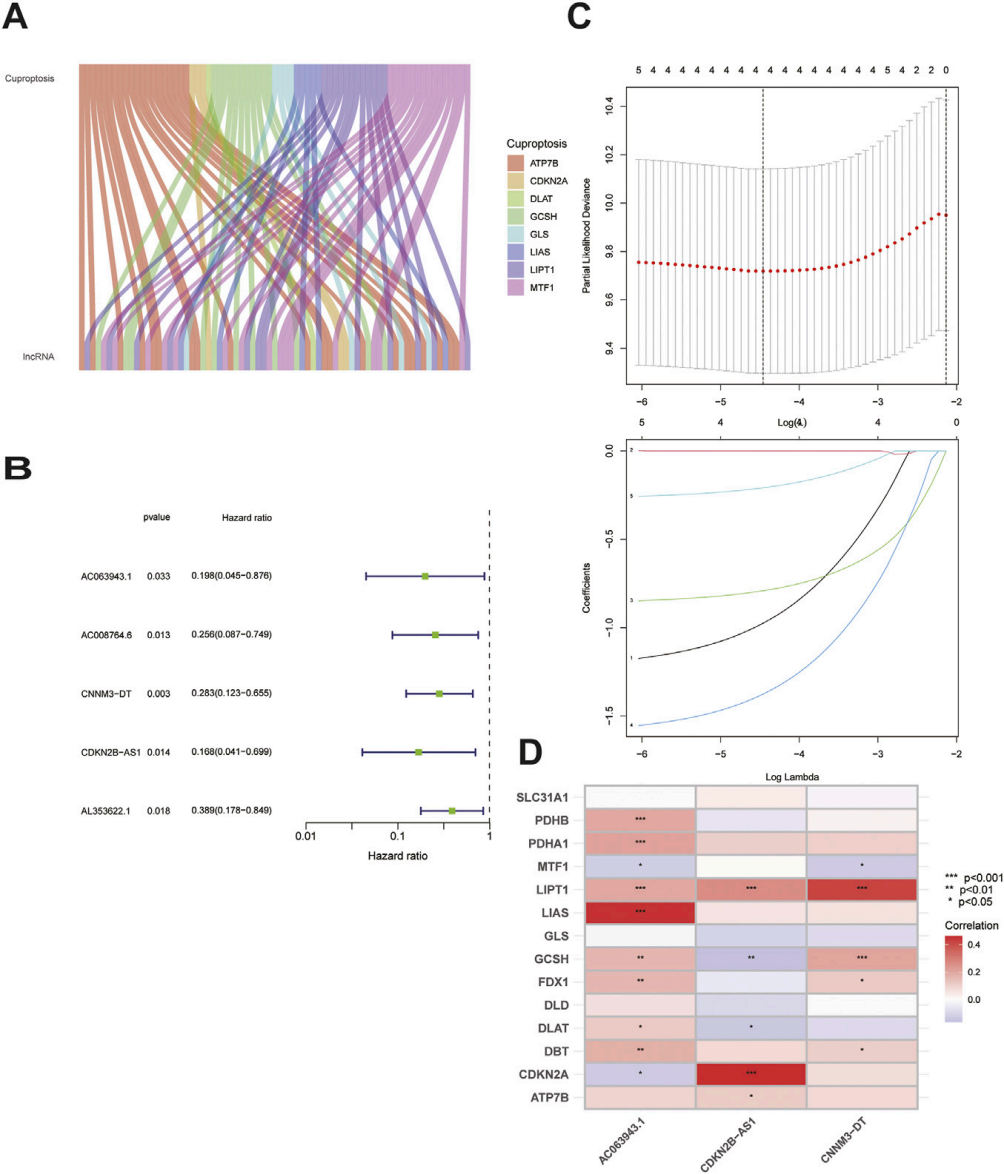
Identification of the signature of cuproptosis-related lncRNA.**(A)**Sankey diagram of 71 lncrnas co-expressed with cuproptosis-related genes.**(B)**Forest plots of 5-lncRNA screening by COX regression related with cuproptosis.**(C)**Lasso regression of the 5-lncRNA.**(D)**Heatmap shows the co-expression relationship between cuproptosis-related lncRNA signature and cuproptosis-related genes.

Our study included 304 patients with CC who were randomly divided into either the training (*n* = 152) or testing (*n* = 152) set in a 1:1 ratio. The clinical and pathological characteristics of all patients are shown in Table 1. There were no statistically significant differences between the training and testing sets in the clinical characteristics based on a clinical statistical analysis of the groups. A total of 14 genes associated with cuproptosis were obtained upon review of the literature, and 71 cuproptosis-related lncRNAs associated with the 14 genes were screened using Pearson’s correlation analysis (|corFilter = 0.4|; pvalueFilter = 0.001) (Figure 1A). Our training set revealed five cuproptosis-related lncRNAs upon univariate Cox regression analysis, and the resultant risk values were presented as forest plots (Figure 1B). Reduced multicollinearity was achieved by performing the Lasso regression analysis, and subsequent multivariate Cox regression analysis allowed us to screen three lncRNAs associated with cuproptosis (AC063943.1, CDKN2B−AS1, and CNNM3–DT; risk score = −AC063943.1 × 1.37125 − CNNM3-DT0.94343-CDKN2B-AS1 × 1.69231), which were verified using the testing set (Figure 1C). As shown through the heat map of the cuproptosis-related genes, the three lncRNAs were positively correlated with LIAS, CDKN2A, and LIPT1, respectively (Figure 1D).

**TABLE 1:** Clinical characteristics of CESC patients involved in the study.

		Total N = 304	Training set n = 152	Testing set n = 152	*p*-value
Age	≤65	269 (88.49%)	133 (87.5%)	136 (89.47%)	0.7193
	>65	35 (11.51%)	19 (12.5%)	16 (10.53%)	
Grade	G1	18 (5.92%)	8 (5.26%)	10 (6.58%)	0.5986
	G2	135 (44.41%)	71 (46.71%)	64 (42.11%)	
	G3	118 (38.82%)	56 (36.84%)	62 (40.79%)	
	G4	1 (0.33%)	0 (0%)	1 (0.66%)	
	unknow	32 (10.53%)	17 (11.18%)	15 (9.87%)	
Stage	Stage I	162 (53.29%)	75 (49.34%)	87 (57.24%)	0.1684
	Stage II	69 (22.7%)	36 (23.68%)	33 (21.71%)	
	Stage III	45 (14.8%)	24 (15.79%)	21 (13.82%)	
	Stage IV	21 (6.91%)	15 (9.87%)	6 (3.95%)	
	unknow	7 (2.3%)	2 (1.32%)	5 (3.29%)	
T	T1	140 (46.05%)	64 (42.11%)	76 (50%)	0.1009
	T2	71 (23.36%)	41 (26.97%)	30 (19.74%)	
	T3	20 (6.58%)	10 (6.58%)	10 (6.58%)	
	T4	10 (3.29%)	8 (5.26%)	2 (1.32%)	
	unknow	63 (20.72%)	29 (19.08%)	34 (22.37%)	
M	M0	116 (38.16%)	59 (38.82%)	57 (37.5%)	0.7417
	M1	10 (3.29%)	4 (2.63%)	6 (3.95%)	
	unknow	178 (58.55%)	89 (58.55%)	89 (58.55%)	
N	N0	133 (43.75%)	64 (42.11%)	69 (45.39%)	0.6066
	N1	60 (19.74%)	32 (21.05%)	28 (18.42%)	
	unknow	111 (36.51%)	56 (36.84%)	55 (36.18%)	

### Characteristics of cuproptosis-related lncRNAs

**FIGURE 2:**
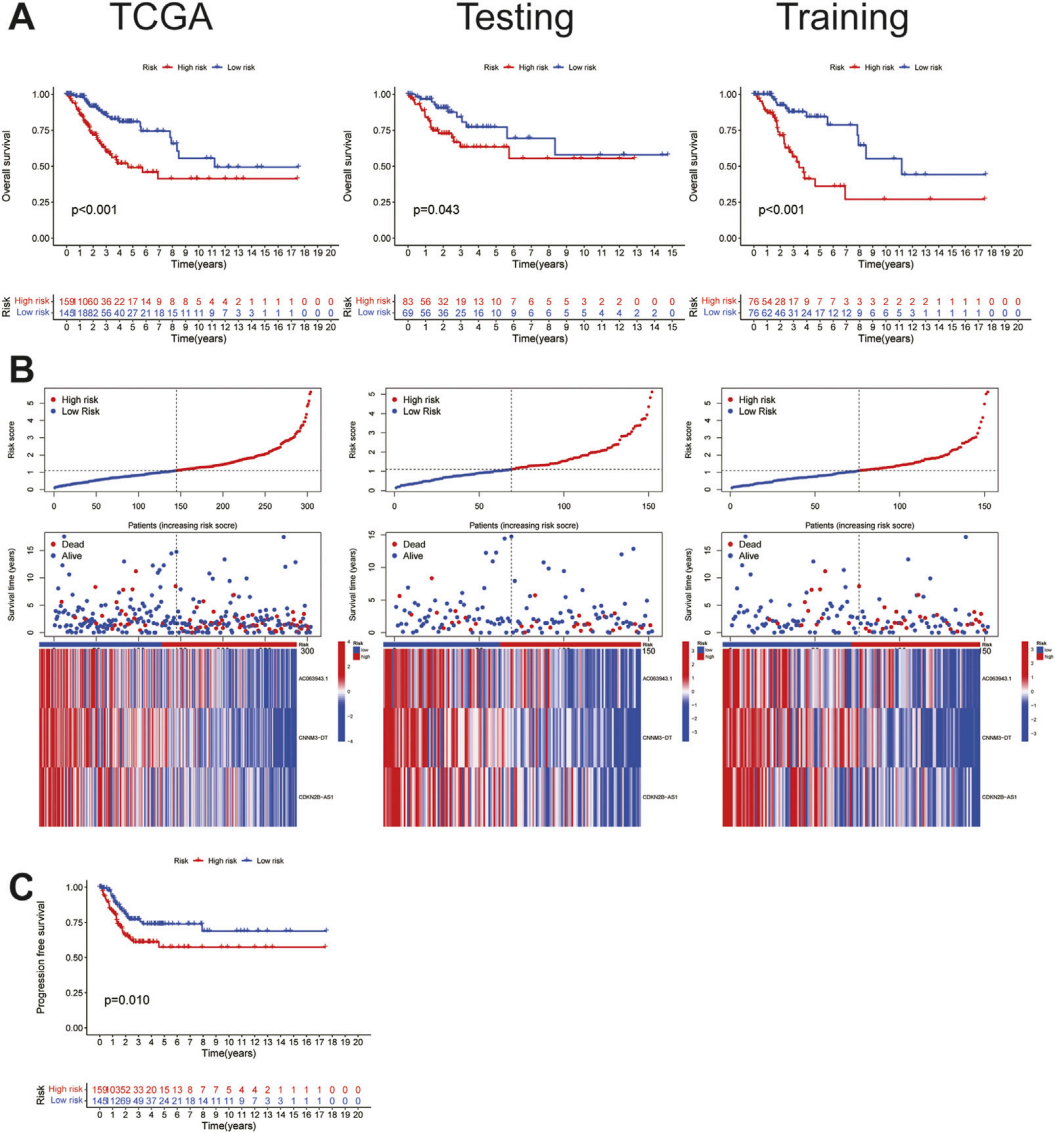
Characteristics of cuproptosis-related lncRNA signature.**(A)**The OS survival analysis of TCGA(risk) set, the training set, and the testing set. **(B)** Risk characteristics between high and low risk groups in TCGA(risk) set, the training set, and the testing set.**(C)** The PFS survival analysis in TCGA(risk) set.

To study the prognostic and risk verification abilities of the three lncRNAs, the risk score of each individual in the testing set was calculated using the same calculation as that used in the training set. Then, we divided all the patients into the HRG and LRG based on the same cut-off values used for the training set, which were verified in each of the three cohorts, including the TCGA, training, and testing sets. Established cuproptosis-related lncRNAs had a strong ability to predict prognosis, including OS (Figure 2A) and PFS (Figure 2B), whereas the patients with elevated risk scores showed higher mortality and worse survival; moreover, the lower the expression of these three lncRNAs, the higher the risk rate, suggesting that these three lncRNAs function as tumor suppressor genes and can help predict the risk among patients. This conclusion was consistently validated in all three sets (Figure 2C).

**FIGURE 3:**
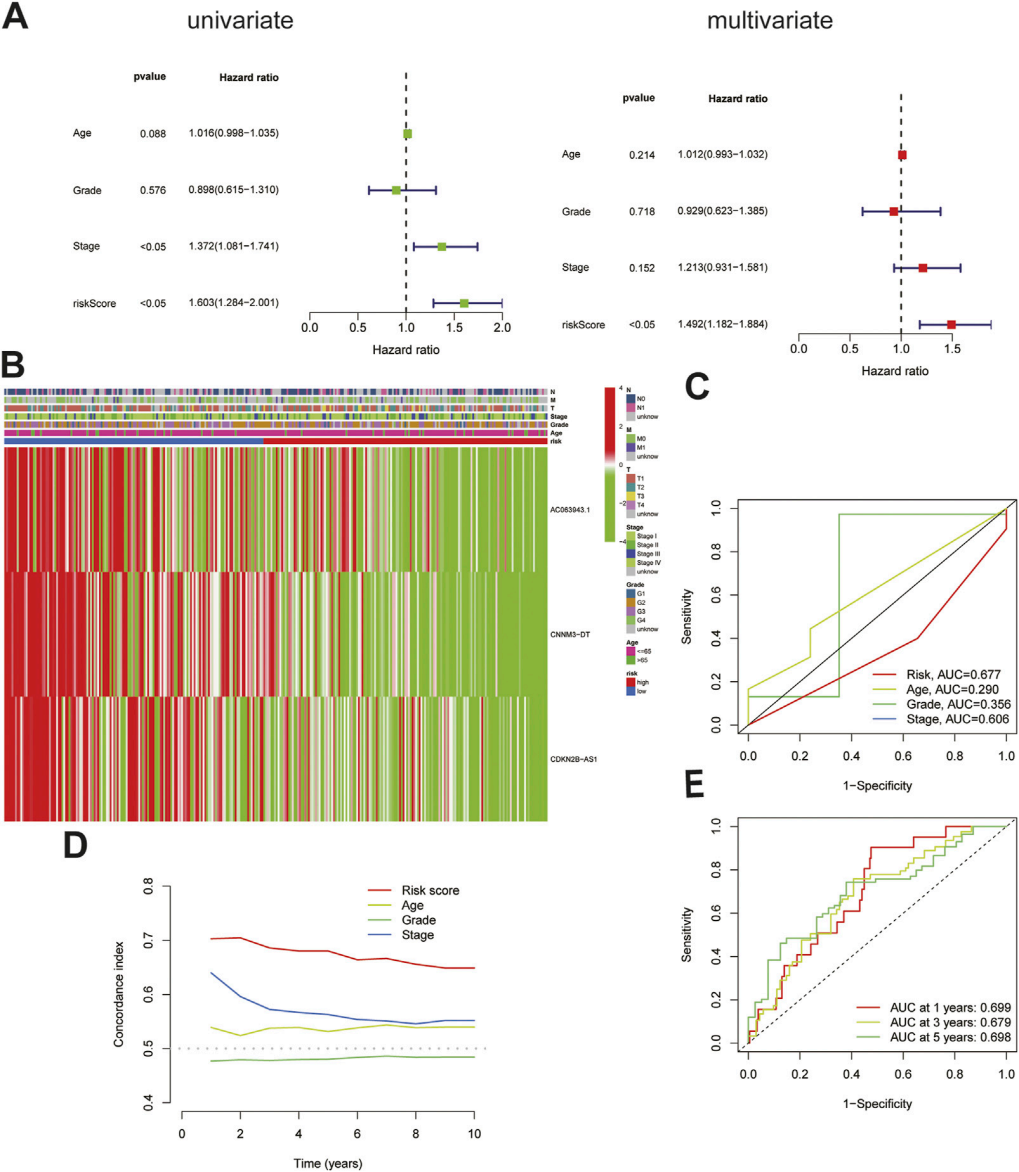
Validation of the cuproptosis-related lncRNA signature. **(A)**The cuproptosis-related lncRNA signature was shown to be an independent risk factor for patients' overall survival in TCGA.**(B)**The heatmap shows the expression of signature in the high and low risk group and their relationship with other clinicopathological signatures.**(C)**The AUC showed that riskscore was an independent predictor compared with other clinicopathological signatures.**(D)**The C-Index of the riskscore was higher than other clinicopathological signatures.**(E)**The signature could be used as an independent predictor to predict the OS of 1-,3-,5 years.

To further verify the accuracy of the cuproptosis-related lncRNA signatures, an independent prognostic analysis was performed among the three cuproptosis-related lncRNAs involved in the construction of the signature and other clinical signatures with complete information. The results of univariate proved that the 3-lncRNAs signature is an independent predictor (Figure 3A) as compared with the other clinical signatures, and the association of the risk prediction model with the other clinical signatures in all CC samples is shown in the heat map (Figure 3B). Furthermore, the ROC curve indicated that the predictive power of this signature relative to other clinical signatures was high, with AUC = 0.677 (Figure 3C). According to the curve, the C-index of this signature was much higher than that of the other three clinical signatures (Figure 3D), and according to the time ROC analysis, the 1-, 3-, and 5-year AUCs of this signature were 0.699, 0.679, and 0.698, respectively (Figure 3E), suggesting that this signature can function as an independent predictor independent of the patients’ age as well as tumor grade and stage and has stronger predictive power and higher confidence as compared with the other clinical signatures, similar to that noted for prognosis.

### PCA and construction of a nomogram to predict patient survival

**FIGURE 4:**
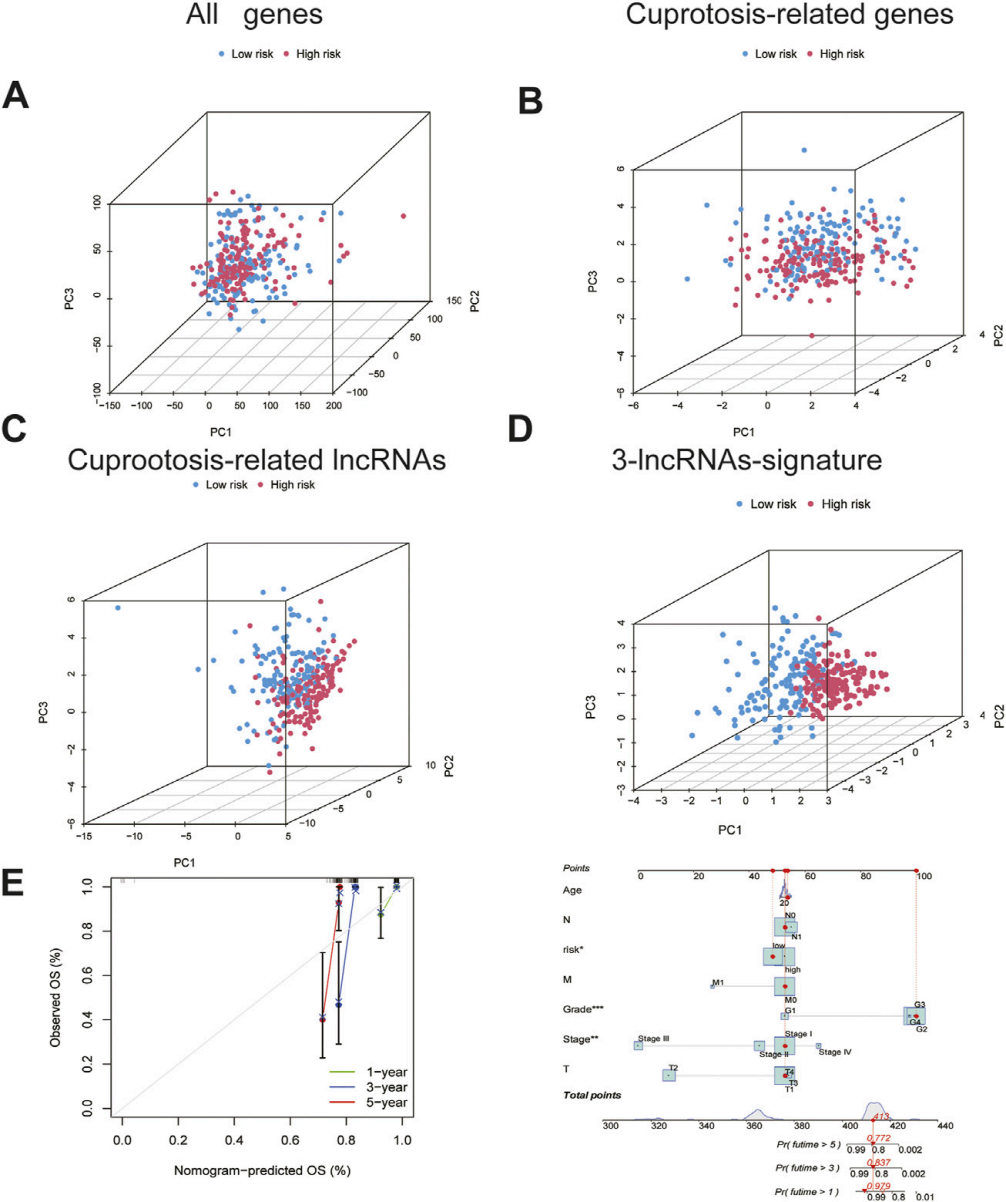
The PCA and nomogram of the signature.**(A)**. PCA of all genes.**(B)**PCA of cuproptosis-related genes. **(C)**PCA of cuproptosis-related lncRNAs.**(D)**PCA of 3-lncRNA signature (risk DE-lncRNAs).**(E)** The nomogram of 3-lncRNA signature.

Differences between the HRG and LRG in terms of all the genes (Figure 4. A), 14 genes associated with cuproptosis (Figure 4B), 71 cuproptosis-related lncRNAs (Figure 4C), and three cuproptosis-related lncRNAs of the signature risk DE-lncRNAs (Figure 4D) were determined using PCA with the ‘scatterplot3d’ R package. The range of gene expression between the HRG and LRG by the 3-lncRNAs signature was relatively well-defined and more predictive than all the genes, 14 genes associated with cuproptosis and 71 cuproptosis-related lncRNAs.

Based on the univariate and multivariate Cox analyses, we analysed the independent prognostic value of the cuproptosis score for the patients with CC as well as the prognostic value of multiple clinical factors. The risk score and clinicopathological features were first combined, after which we developed a mixed nomogram to predict the 1-, 3-, and 5-year survivals because the cuproptosis-related lncRNA risk score is of limited clinical value in predicting OS in patients with CC owing to its inconvenient clinical utility. Predictors included risk score, age, tumor grade, and stage. In subsequent calibration plots, the proposed model was shown to be closer to the ideal model (Figure 4E).

### Relationship between the characteristics of cuproptosis-related lncRNAs and radiotherapy

**FIGURE 5:**
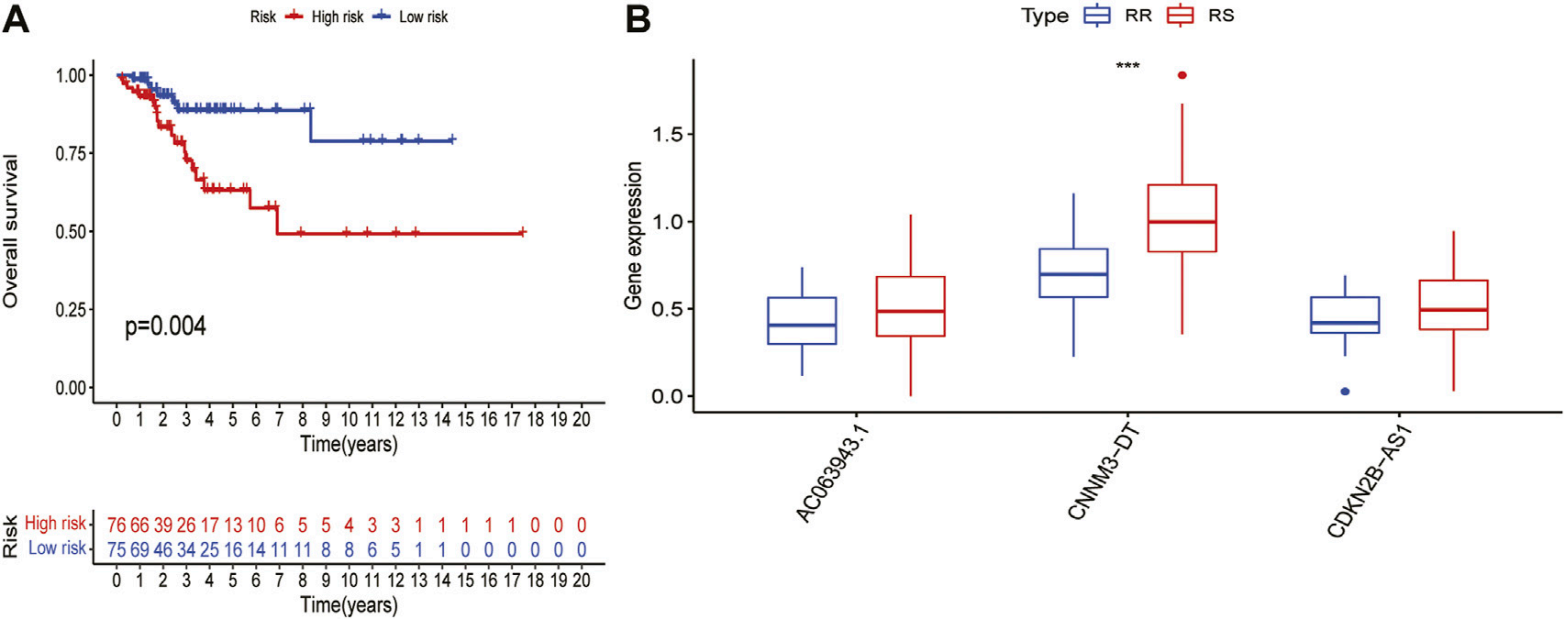
Prediction of risk score in patients with cervical cancer undergoing radiotherapy. **(A)**Differences in OS between HRG - and LRG in patients receiving radiotherapy. **(B)**Differences in expression of three lncRNAs in the signature between RS and RR groups.

For patients with advanced CC, the main treatment is chemoradiotherapy, especially radiotherapy. To study the relationship between this signature and radiotherapy, of the 304 patients from TCGA dataset, we found 151 patients labelled to receive radiotherapy. The 151 patients were divided into two different groups according to the risk score. There was no significant difference in the clinical characteristics between the two groups Table 2. Compared with that in the LRG, the OS was significantly worse in the HRG (*p* = 0.004; Figure5.A), indicating that this signature had a certain predictive effect on the prognosis of the patients undergoing radiotherapy. However, the prognosis of these patients was mainly determined by their sensitivity to radiotherapy, indicating that this signature may affect the sensitivity of the patients undergoing radiotherapy to a certain extent, resulting in differences in the prognosis. A total of 73 patients with a complete radiotherapy evaluation were further evaluated for their complete response and partial response by radiotherapy and classified into the radiosensitive (RS) group; by contrast, those evaluated for progressive disease (PD) and stable disease (SD) were classified into the radiotherapy resistance group (RR) group Table 3. We investigated the differential expression of three lncRNAs in this signature between RS and RR groups but found no significant expression differences in AC063943.1(*p* = 0.176) and CDKN2B-AS1(*p* = 0.174); however, CNNM3-DT was differentially expressed between the two groups (*p* < 0.001), indicating that CNNM3-DT may be a target affecting radiosensitivity in patients (Figure 5B).

**TABLE 2:** Clinical characteristics of patients who received radiotherapy.

Covariates	Type	Total	Low	High	*p*-value
Age	≤65	133 (88.08%)	66 (88%)	67 (88.16%)	1
	>65	18 (11.92%)	9 (12%)	9 (11.84%)	
Grade	G1	6 (3.97%)	3 (4%)	3 (3.95%)	0.7879
	G2	66 (43.71%)	33 (44%)	33 (43.42%)	
	G3	64 (42.38%)	33 (44%)	31 (40.79%)	
	G4	1 (0.66%)	0 (0%)	1 (1.32%)	
	unknow	14 (9.27%)	6 (8%)	8 (10.53%)	
Stage	Stage I	71 (47.02%)	36 (48%)	35 (46.05%)	0.5486
	Stage II	43 (28.48%)	24 (32%)	19 (25%)	
	Stage III	26 (17.22%)	10 (13.33%)	16 (21.05%)	
	Stage IV	7 (4.64%)	3 (4%)	4 (5.26%)	
	unknow	4 (2.65%)	2 (2.67%)	2 (2.63%)	
T	T1	56 (37.09%)	30 (40%)	26 (34.21%)	0.7078
	T2	44 (29.14%)	23 (30.67%)	21 (27.63%)	
	T3	11 (7.28%)	5 (6.67%)	6 (7.89%)	
	T4	4 (2.65%)	1 (1.33%)	3 (3.95%)	
	unknow	36 (23.84%)	16 (21.33%)	20 (26.32%)	
M	M0	53 (35.1%)	27 (36%)	26 (34.21%)	1
	M1	7 (4.64%)	4 (5.33%)	3 (3.95%)	
	unknow	91 (60.26%)	44 (58.67%)	47 (61.84%)	
N	N0	54 (35.76%)	29 (38.67%)	25 (32.89%)	0.6035
	N1	35 (23.18%)	16 (21.33%)	19 (25%)	
	unknow	62 (41.06%)	30 (40%)	32 (42.11%)	

**TABLE 3:** Clinical characteristics of patients who received radiotherapy and had complete radiotherapy evaluation.

		Patients of radiotherapy (N = 73)
Age	≤65	65
	>65	8
T	T1	23
	T2	22
	T3	13
	T4	4
	Unkown	11
Stage	stageⅠ	24
	stageⅡ	23
	stageⅢ	14
	stageⅣ	9
	Unkown	3
G	G1	2
	G2	34
	G3	25
	Unkown	12
Measure of response	CR	51
	PR	7
	PD	13
	SD	2

### Analysis of functional enrichment

**FIGURE 6:**
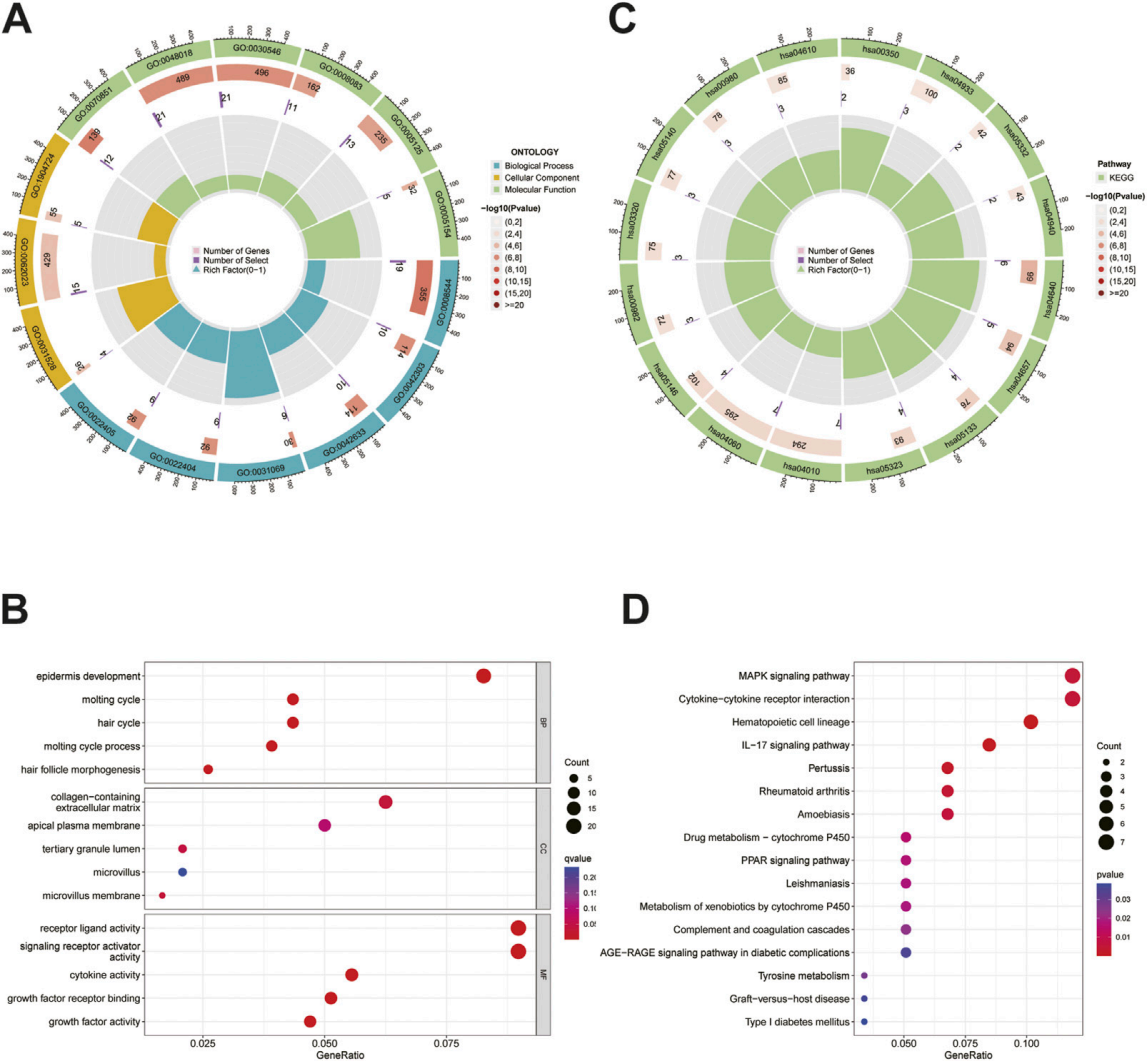
GO and KEGG analysis. **(A)**The circle graph of GO analysis. **(B)**The bubble diagram of GO analysis. **(C)**The circle graph of KEGG analysis. **(D)**The bubble diagram of KEGG analysis.

The differential genes between the HRG and LRG were analyzed using GO and KEGG analyses to investigate the functions and pathways enriched by this signature. The results of GO analysis indicated that the molecular functions of these genes were mainly enriched in terms of cell–cell signals exchange pathways as well as the biological processes mediating immunity, such as ligand-receptor activity and activator receptor signaling activity (Figure 6A-B). Significant enrichment of genes encoding MAPK signalling pathways and the interaction between cytokine receptors and the cytokines found by KEGG pathways, showing that these genes were closely associated with immune function (Figure 6C-D).

### Differences in tumor immune infiltration

**FIGURE 7:**
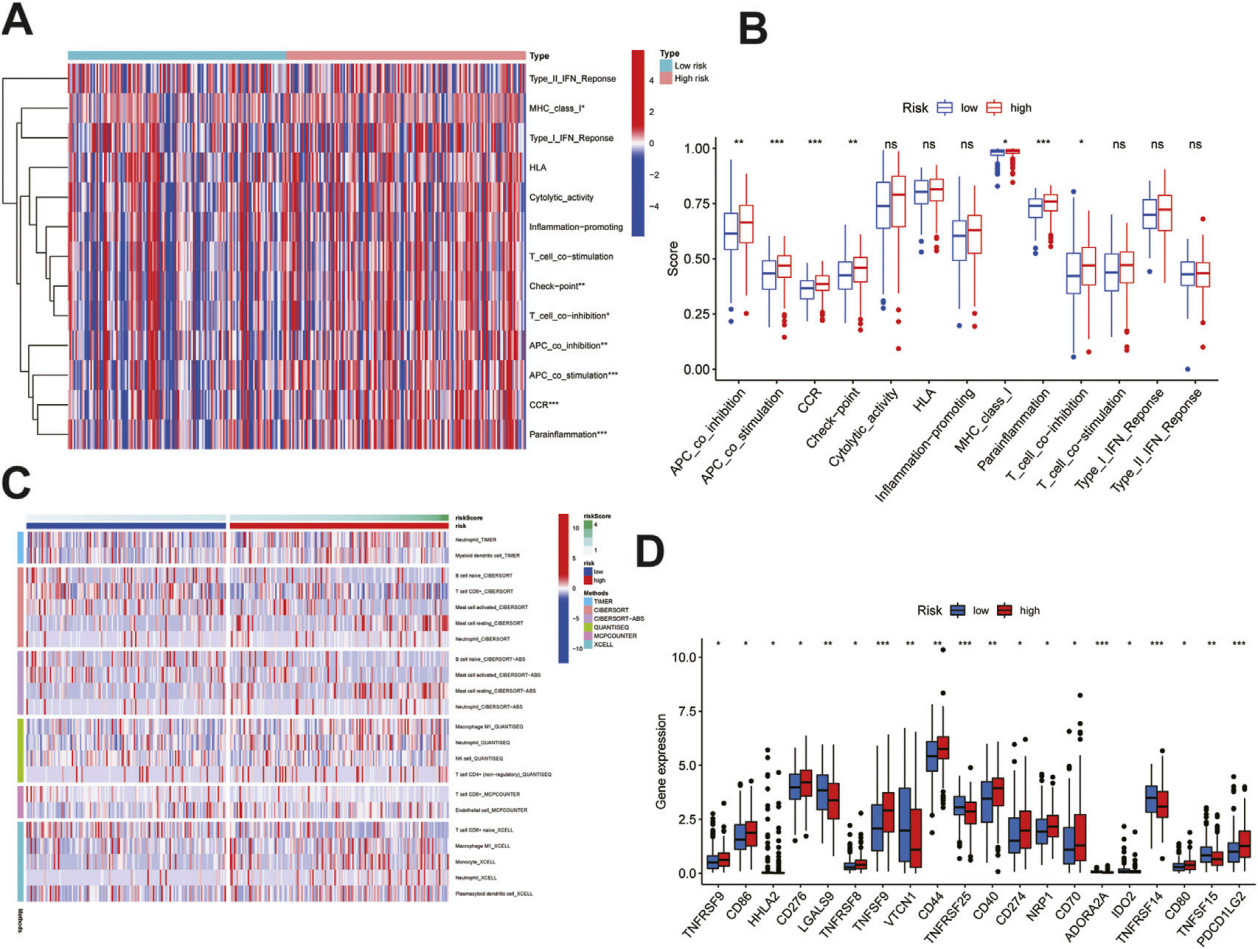
Immune infiltration analysis of the signature. **(A)**Heatmap showed immune cells and immune function between high and low risk groups in all samples. **(B)**Differences in immune function between high and low risk groups. **(C)**Heatmap of immune penetration based on TIMER, CIBERSORT, CIBERSORT-ABS, QUANTISEQ, MCPCOUNTER, and XCELL algorithms. **(D)**Differences in immune checkpoints between high-and low-risk groups. Data are shown as means ± S.D. ns: not significant, *p < 0.05, **p < 0.01, ***p < 0.001.

According to the functional enrichment results, the genes involved in immune pathways were more prominent in the HRG than in the LRG; therefore, we continued to study the relationship between the risk score and tumor immune microenvironment. First, in terms of immune function and immune cells, the most significant differences were in terms of APC co-stimulation, CCR, parainflammation, APC co-inhibition, checkpoint, MHC class-I, and T-cell co-inhibition; these parameters were also different between the two groups and were positively associated with risk (Figure 7A–B). Immune penetration is represented by a heat map constructed using the TIMER, CIBERSORT, CIBERSORT-ABS, QUANTISEQ, MCPCOUNTER, and XCELL algorithms (Figure 7C). Given the importance of checkpoint-based immunotherapy, immune cell and functional analyses suggested that the immune checkpoints were different in the HRG and LRG, and further differences in the immune checkpoint expression between the two groups were found and studied further. Many immune checkpoint inhibitors, including CD274, were found to be differentially expressed between the two groups (Figure 7D). The most significant differences were found for TNFSF9, TNFRSF25, ADORA2A, TNFRSF14, and PDCD1LG2. Based on these results, significant differences were noted in the immune infiltration, including immune cells, function, and checkpoints, between the two different risk groups.

### Risk score-based tumor mutational landscape and prognostic differences in CC

**FIGURE 8:**
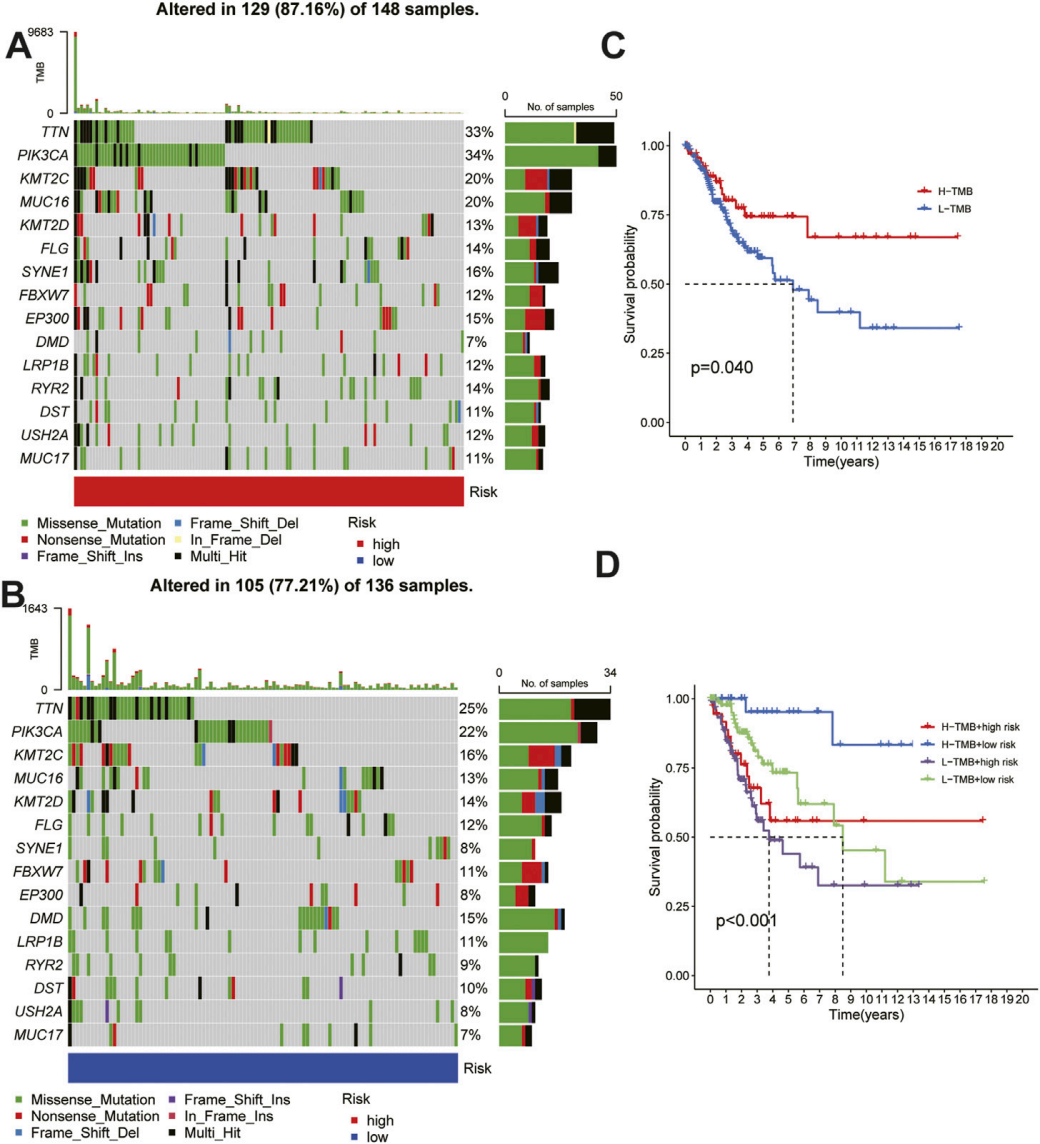
Relationship between risk score and tumor mutation burden. **(A) (B)** Waterfall plots of somatic mutation characteristics in the high-risk and low risk groups.**(C)**K–M survival curves between the high- and low-TMB groups. **(D)**K–M survival curves between the four groups.

To explore the differences between HRG and LRG in terms of tumor mutations, we studied the somatic mutation frequencies between the two groups and identified the top 15 genes with the highest mutation rates. A significant difference was observed between the HRG (87.16%) and the LRG (77.21%) in terms of the total mutation rate (Figure 8A–B); the mutation rate of the 14 genes was higher in the HRG than in the LRG, and only the mutation rate of DMD was lower in the HRG (7%) than in the LRG (15%). Moreover, patients with different tumor mutation burdens also had different survival rates (Figure 8C), and the patients with high mutation rates had higher survival rates than those with low mutation rates. We further compared the survival rates of patients after a combined analysis of the risk score and tumor mutation burden, and patients with a higher tumor mutation burden (TMB) in the LRG were found to have the highest survival rate, whereas those with a lower TMB in the HRG had the lowest survival rate (Figure 8D).

## Discussion

Approximately 111,820 new cases of CC and 61,579 deaths due to CC will occur in China (including Taiwan) in 2022 (Xia et al., 2022). In the United States, the number of new CC cases has significantly decreased owing to CC vaccines and screening, however, since the 1970s, CC is the only cancer type of which the survival rate has not improved significantly (Li et al., 2016; Siegel et al., 2018). The 5-year survival rate of patients with metastatic, recurrent, or persistent CC is 20%, and only limited treatment options are available (Pfaendler and Tewari, 2016; Siegel et al., 2018). Radiotherapy/chemoradiotherapy and immunotherapy are the most important treatment options; moreover, although there have been many new advances in immunotherapy for CC, there are already several drug combinations, such as sintilimab + anlotinib, that can be used to treat PD-L1-positive recurrent or metastatic CC (Xu et al., 2022b). Plant-derived natural products are also available as antitumor immunotherapy agents (Yang et al., 2021). So far, only one immunotherapy drug (pembrolizumab) has been approved by the FDA for CC treatment (Ferrall et al., 2021). Radiotherapy and chemotherapy have certain side effects and adverse reactions, and whether it is beneficial to the prognosis of patients depends on their sensitivity to radiotherapy.

Recently, Tsvetkov et al. identified copper disease as a novel apoptotic process with dual functions in tumor development and treatment, and the development of this disease helped kill malignant cells and remove defective cells by overcoming their resistance to chemotherapy (Lu et al., 2022). In the future, copper trichomoniasis could be a promising treatment option for various cancers (Xu et al., 2022c). Additionally, lncRNAs biologically impact cancer development. Therapeutic targeting of non-coding RNAs, especially lncRNAs, represents an attractive approach for the treatment of cancers and other diseases. (Winkle et al., 2021). LncRNAs were found to play an important role in the cellular biological behavior of CC through various mechanisms, and these molecules may be effective molecular targets in the treatment of CC (Yang and Al-Hendy, 2022). Thus, in the future, we should study the potential interactions between lncRNAs and the activity of copper to identify potential prognostic markers and to find the predicted and therapeutic targets of CC.

In this study, we obtained a three-lncRNA-signature and assessed the risk score. According to the risk score, the patients with CC were divided into HRG and LRG, which were statistically significantly different in terms of OS and PFS. At the same time, the signature was more predictive than the clinicopathological factors of CESC. The ROC curve confirmed its favorable prediction validity for the 1-, 3-, and 5-year operating systems. Risk analysis, PCA, and nomogram analysis also confirmed this conclusion. The prediction model based on the risk score could better distinguish between the HRG and LRG and showed an effective prediction ability. In the clinical setting, transcriptome sequencing can be performed for pathological samples of patients with CC to determine the patient’s risk scores. High-risk scores were associated with shorter OS and PFS, indicating that high-risk scores may predict adverse outcomes and that their predictive power is reliable. Furthermore, whether lncRNAs influence the development, radiosensitivity, and prognosis of patients with CC through cuproptosis-related regulatory mechanisms remains largely unknown. We investigated whether the risk signature could help predict the outcomes of the patients undergoing radiotherapy and the possible targets affecting radiotherapy sensitivity. Among the 151 patients in the TCGA–CESC cohort who were identified as having received radiotherapy, the predictive ability of the signature for the patient’s outcomes was highlighted. When the 73 patients with complete efficacy evaluation were studied further, CNNM3-DT was the only lncRNA that was differentially expressed between the RS and NRS groups. This lncRNA may also function as a target, however, this has never been reported to date. We hope to further confirm this in future research and study the mechanisms underlying the association between cuproptosis and radiation sensitivity.

The functional enrichment analysis results revealed the biological mechanisms underlying the three lncRNA signatures involved. We identified 173 differentially expressed genes between HRG and LRG. The GO and KEGG analysis results showed that these differentially expressed genes were mainly enriched in terms of signal transduction and immune-related pathways, suggesting that cuproptosis may affect intercellular signaling; for example, cytokine–cytokine receptor interaction is a major contributing factor to cellular inflammation. (Karlsson et al., 2021).

The immune response plays a primary role in tumorigenesis and can often be used as a target for tumor therapy. Related function analysis of immune cell subsets showed that APC co-inhibition, stimulation, CCR, immune checkpoint, MHC class I, parainflamation, and T-cell co-inhibition were enhanced in the HRG, suggesting that elevated tumor immunity may lead to poor prognosis. Therefore, promoting antitumor immune responses is essential to prevent the further development of CC at an early stage and to generate effective clinical treatments. In the analysis of immune cells, CD8^+^ T-cell expression was significantly lower in the HRG, and previous studies have shown that CD8^+^ T-cell exhaustion leads to cancer progression (Dolina et al., 2021), suggesting that cuproptosis is associated with CD8^+^ T-cell exhaustion. In addition, in the analysis of the differences in immune checkpoints between the two groups, as members of TNF receptors (TNFRSF), TNFRSF25, TNFSF15, and TNFRSF14 were found to regulate B and T-cell activation, promote dendritic cell proliferation, and protect the mucosal epithelium from damage during inflammation. Therefore, TNFRSF has an inhibitory effect on cancer (Vanamee and Faustman, 2020), which is supported by our finding that its expression is increased in the LRG. ADORA2A also showed differential expression; however, its expression level was low, and it was difficult to determine the relationship between ADORA2A and the risk score for the time being. As another member of the TNF family, the levels of TNFSF9 were increased in the HRG, which was inconsistent with that reported in previous studies. However, all of these findings need to be further verified and discussed in future studies. PD-L1 and CTLA-4 antibody therapy are the main regimens (Dyer et al., 2021) used in CC immunotherapy. CD274 (PDL-1) expression was increased in the HRG; the expression of CD80 and CD86, as ligands of CTLA-4, was also increased in the HRG, and these findings are consistent with those of previous studies (Wendel Naumann and Leath, 2020). This indicates that the HRG and LRG distinguished using this prediction model could be used as potential immunotherapeutic targets. Clinically, sequencing of patients receiving immunotherapy by grouping them according to this risk score can predict the effect of immunotherapy.

Mutations in cancer-causing genes are significantly associated with cancer, and CESC patients with high TMB have better survival outcomes (Wen et al., 2021), consistent with our results. Combined analysis of high- and low-risk scores and TMB in the HRG and LRG showed that patients with high TMB combined with low-risk scores had a significantly higher survival rate. Accordingly, the signature may have a strong predictive ability for the prognosis of CESC patients, especially in combination with the TMB.

Among the three lncRNAs in our signature, CDKN2B–AS1 was confirmed to be involved in the occurrence and development of various diseases, lipid metabolism, and carbohydrate metabolism. It was also found to be involved in the regulation of inflammation, particularly in tumors and various inflammatory diseases (Song et al., 2020). The research about AC063943.1 and CNNM3–DT has not been reported to date, and this will be one of our research goals in the future.

We explored the biomarkers of lncRNA associated with cuproptosis, which could be used for the prognosis of CC, and provided information for the treatment of this disease. Despite this, there are still many shortcomings in our research. Firstly, our research data comes from the TCGA database. The information in public databases has its limitations, such as racial differences and incomplete information. After that, it is necessary to validate the signature in a large multicenter cohort, especially since fewer complete radiotherapy samples are recorded in TCGA, and validation of large samples is required for the impact of our characteristics on radiotherapy sensitivity. Moreover, for the validation of our findings and to uncover the mechanism of action in CC, further functional experiments are needed in our laboratory.

## Conclusion

We have identified a prognostic signature of three cuproptosis-related lncRNAs that has been proven to be independent and highly reliable. By comprehensive analysis, the finding of our study revealed potential biomarkers and therapeutic targets for cuproptosis-related signatures in cervical cancer.

## Data Availability

The original contributions presented in the study are included in the article/supplementary materials, further inquiries can be directed to the corresponding author.

## Abbreviations

CESC, Cervical squamous cell carcinoma and endocervical adenocarcinoma; CC, Cervical cancer; WHO, World HealthOrganization; TCGA, The Cancer Genome Atlas; LASSO, Least absolute shrinkage and selection operator; OS, Overall survival; PFS, Progression-free survival; GSEA, Gene Set Enrichment Analysis; HR,Hazard ratio; HRG, High-risk group; LRG, Low-risk group; CR, Complete response; PR, partial response; PD, progressive disease; SD, stable disease; K-M, Kaplan–Meier; GO, Gene ontology; RS, Radiosensitivity; RR, Radiotherapy resisdance.
